# Characterization of local and circulating bovine γδ T cell responses to respiratory BCG vaccination

**DOI:** 10.1038/s41598-019-52565-z

**Published:** 2019-11-05

**Authors:** Mariana Guerra-Maupome, Jodi L. McGill

**Affiliations:** Department of Veterinary Microbiology and Preventive Medicine, Iowa State University, Ames, IA USA

**Keywords:** Innate immune cells, Lymphocytes

## Abstract

The *Mycobacterium bovis* Bacillus Calmette-Guerin (BCG) vaccine is administered parenterally to infants and young children to prevent tuberculosis (TB) infection. However, the protection induced by BCG is highly variable and the vaccine does not prevent pulmonary TB, the most common form of the illness. Until improved TB vaccines are available, it is crucial to use BCG in a manner which ensures optimal vaccine performance. Immunization directly to the respiratory mucosa has been shown to promote greater protection from TB in animal models. γδ T cells play a major role in host defense at mucosal sites and are known to respond robustly to mycobacterial infection. Their positioning in the respiratory mucosa ensures their engagement in the response to aerosolized TB vaccination. However, our understanding of the effect of respiratory BCG vaccination on γδ T cell responses in the lung is unknown. In this study, we used a calf model to investigate the immunogenicity of aerosol BCG vaccination, and the phenotypic profile of peripheral and mucosal γδ T cells responding to vaccination. We observed robust local and systemic *M. bovis-*specific IFN-γ and IL-17 production by both γδ and CD4 T cells. Importantly, BCG vaccination induced effector and memory cell differentiation of γδ T cells in both the lower airways and peripheral blood, with accumulation of a large proportion of effector memory γδ T cells in both compartments. Our results demonstrate the potential of the neonatal calf model to evaluate TB vaccine candidates that are to be administered via the respiratory tract, and suggest that aerosol immunization is a promising strategy for engaging γδ T cells in vaccine-induced immunity against TB.

## Introduction

*Mycobacterium bovis* is a member of the *M. tb* complex and is the causative agent of bovine TB (bTB) and zoonotic TB infection^1^. The attenuated *M. bovis* vaccine strain, Bacille Calmette-Guerin (BCG), is the only vaccine that is currently available to prevent TB infection in humans. It is approved for intradermal use and is commonly administered at birth to infants in TB endemic areas. The BCG vaccine has been tested experimentally in cattle, and like humans, the protection induced by parenteral BCG vaccination is transient and highly variable [reviewed^2^]. Although parenteral BCG vaccination is not efficacious against pulmonary TB, no other vaccine has shown improved efficacy over BCG, and it remains the ‘gold-standard’ to which all other TB vaccines are compared in both humans and cattle. Furthermore, BCG has well-recognized health benefits in human infants and will likely continue to be administered to populations in developing countries [reviewed^3^]. Therefore, there is significant interest in investigating alternative routes for BCG vaccination, which may prove more efficacious for the prevention of pulmonary TB.

Immunization directly to the nasal or respiratory mucosa with BCG, attenuated *M. tb* and vectored vaccines has been shown to promote greater protection from TB in rodents and non-human primates^4–10^. In BCG-vaccinated cattle, boosting via endobronchial administration with AdAg85A induces local and systemic responses that are similar in magnitude to intradermal boosting^11,12^. Vaccine-induced protection that is observed after aerosol and endobronchial immunization is believed to be associated with the preferential recruitment of antigen*-*specific CD4 T cells to the lung airways^5,13–18^, which allows for an immediate response upon pathogen exposure, preventing bacilli from establishing infection^19–21^. While it is clear that IFNγ-secreting CD4 T cells are an essential component for protection against TB, IFNγ secretion is a poor correlate of protection^22,23^. The activity of other lymphocyte populations, notably γδ T cells, may represent another major essential component for protection or resistance to TB [reviewed^24^].

γδ T cells play a major role in defense against pathogens, especially at mucosal sites such as the lung^25–28^. Although γδ T cells fall into the innate-like category, adaptive features have also been reported in multiple studies [reviewed^29,30^]. Humans vaccinated parenterally with BCG have a population of γδ T cells that expands robustly in response to *in vitro* restimulation with mycobacteria antigens^31^. In non-human primates, administration of phosphoantigens/IL-2 induced a marked expansion and pulmonary accumulation of phosphoantigen-specific Vγ2Vδ2 T cells, significantly reducing *M. tb* burdens and associated lung pathology^9,32^.

Like CD4 T cells, γδ T cells have the capacity to differentiate into subsets that differ in their migratory and functional properties. In humans, γδ T cell subsets are divided according to the surface expression of CD45RA and CD27. Naïve CD45RA^+^ CD27^+^ cells represent ~10–20% of the γδ T cells circulating population in healthy adults. Central memory (T_CM_) cells CD45RA^−^ CD27^+^ are more plentiful in the blood and exhibit robust proliferative capacity, but limited effector functions^33^. Effector memory (T_EM_) and CD45RA^+^ CD27^−^ (T_EMRA_) γδ T cells are generally recognized to be fully differentiated subsets and express receptors for homing to inflamed tissues, display immediate effector functions and are highly prevalent in sites of inflammation^34^. Consistent with their differential homing capacity, certain chemokine receptors are also useful for classifying functional γδ T cell subsets^35^. The expression of the homing receptors CXCR3, CCR5 and CD62L have been used to differentiate effector and memory γδ T cells subsets^36,37^.

Effector T cells expand during active disease, whereas memory cells correlate with reduced mycobacterial burden and associated pathology following experimental infection^38,39^. Interestingly, serious TB disease results in reduced γδ T cell effector functions in the periphery^33,34^. Consistent with this observation, there is a progressive loss of CD27^neg^ T_EM_ and T_EMRA_ γδ T cell subsets from the peripheral blood of patients with active TB^34,40^. We have recently shown that virulent *M. bovis* infection results in differentiation of circulating bovine γδ T cells to a T_CM_ phenotype similar to that described in humans^41^. However, little is known regarding the response by γδ T cells in the respiratory tract during mycobacterial infection and vaccination^42,43^, and there are limitations for assessing the biological significance of γδ T cells in the response to TB in humans.

As a natural host of TB infection, cattle represent a highly relevant animal model to investigate the immune response of γδ T cells to mycobacterium vaccination and infection^2,44,45^. Furthermore, respiratory BCG vaccination is an established, well-characterized experimental system that is particularly useful for studying the development of TB-specific immune responses in the lungs. Serial bronchoalveolar lavages can be conducted in cattle, which allow longitudinal analysis of the cell populations which are recruited to the lungs following aerosol vaccination, and which have been implicated in promoting increased resistance to TB infection. To this end, calves were vaccinated with BCG via the respiratory tract, and vaccine immunogenicity, and the differentiation of responding *M. bovis*-specific γδ T cells was examined in the peripheral blood and lower respiratory tract. We hypothesized that vaccination with BCG via the respiratory tract would result in the development of robust immune responses and phenotypic changes in local and circulating γδ T cell populations. Improved characterization of γδ T cell phenotype and function during mycobacterial infection and vaccination will contribute to the development of improved strategies for harnessing their response in protection against TB.

## Results

### Aerosol BCG vaccination induced robust local and systemic cellular immune responses

**Figure 1:**
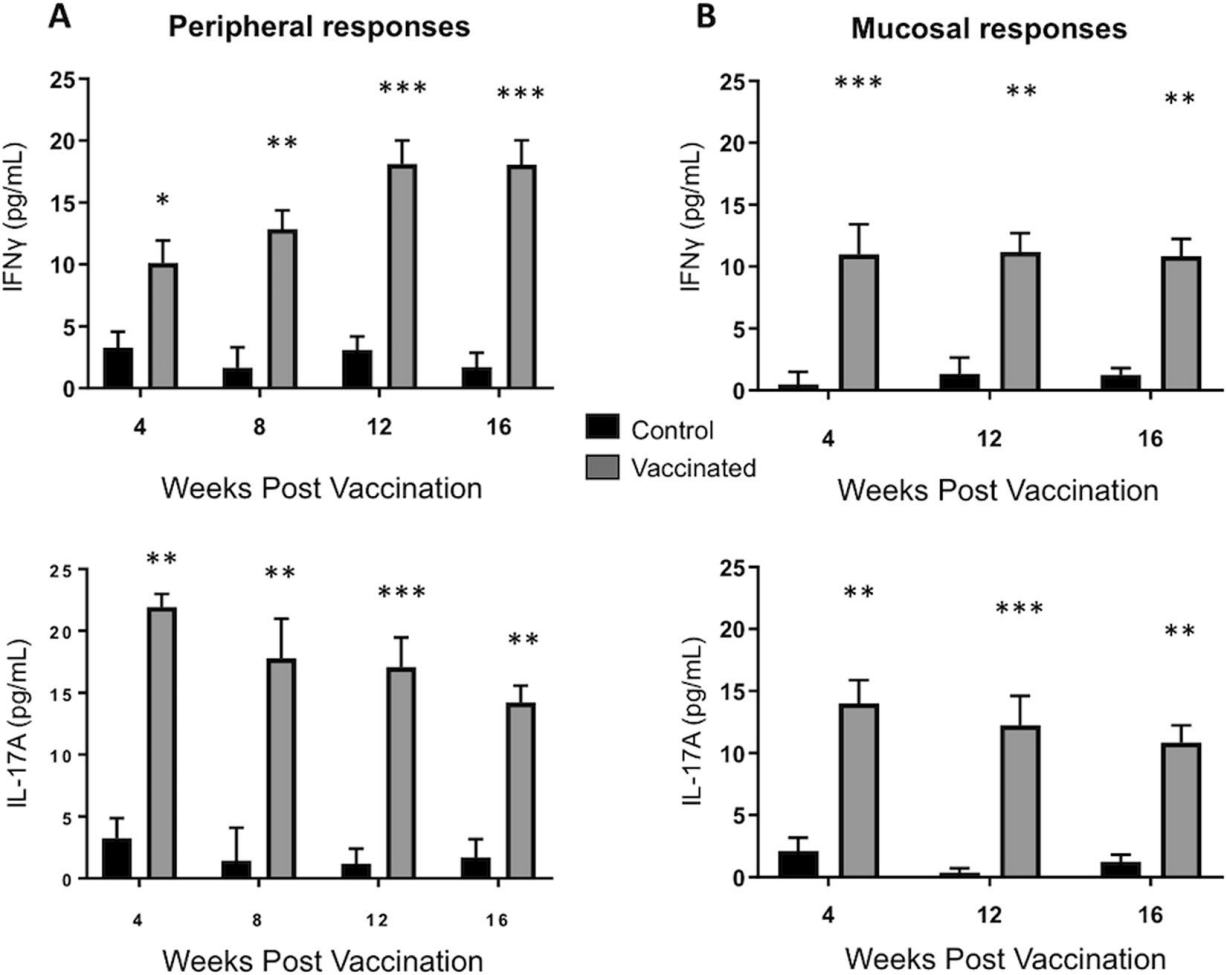
Cellular immune responses in the airways and peripheral blood of BCG-vaccinated calves. (**A**) Peripheral blood was collected 4-, 8-, 12- and 16-weeks after vaccination from control (n = 7) or BCG–vaccinated animals (n = 7). PBMC were isolated and stimulated for 6 days with PPD-b. Control wells remained unstimulated. (**B**) BAL were collected at 4-, 12- and 16-weeks after vaccination. BAL cells were stimulated for 72 hours *in vitro* with PPD-b. Control wells remained unstimulated. Cell culture supernatants were collected from BAL and PBMC cultures and analyzed by commercial ELISA kit for IFNγ (upper panel) and IL-17 (lower panel). Data represent means ± SEM. *p < 0.05, **p < 0.01 ***p < 0.001 as determined by Student’s t test. This figure was previously published^67^ and is duplicated with permission.

One of the primary objectives of this study was to determine the immunogenicity of a BCG vaccine delivered by aerosol to cattle. To this end, calves were vaccinated via aerosol inoculation with BCG. Control calves remained unvaccinated. PBMC were isolated at 4, 8, 12- and 16-weeks post vaccination (Fig. 1A). Cells were restimulated *in vitro* as described in Materials & Methods. IFNγ (upper panels) and IL-17A (lower panels) concentrations were analyzed in cell culture supernatants by sandwich ELISA. Aerosol immunization was highly immunogenic and elicited potent *M. bovis-*specific immune responses in peripheral blood, as indicate by the robust IL-17A and IFNγ secretion by Ag-stimulated PBMC. The response was significantly increased over PBMC from unvaccinated controls throughout the 16-week study (Fig. 1A).

Vaccine-induced responses in the respiratory mucosa were measured from BAL mononuclear cells at 4-, 12- and 16-weeks post vaccination (Fig. 1B). BAL cells from BCG vaccinated calves produced significant quantities of IL-17A and IFNγ in recall response to PPD-b, while BAL cells from unvaccinated animals did not (Fig. 1B).

**Figure 2:**
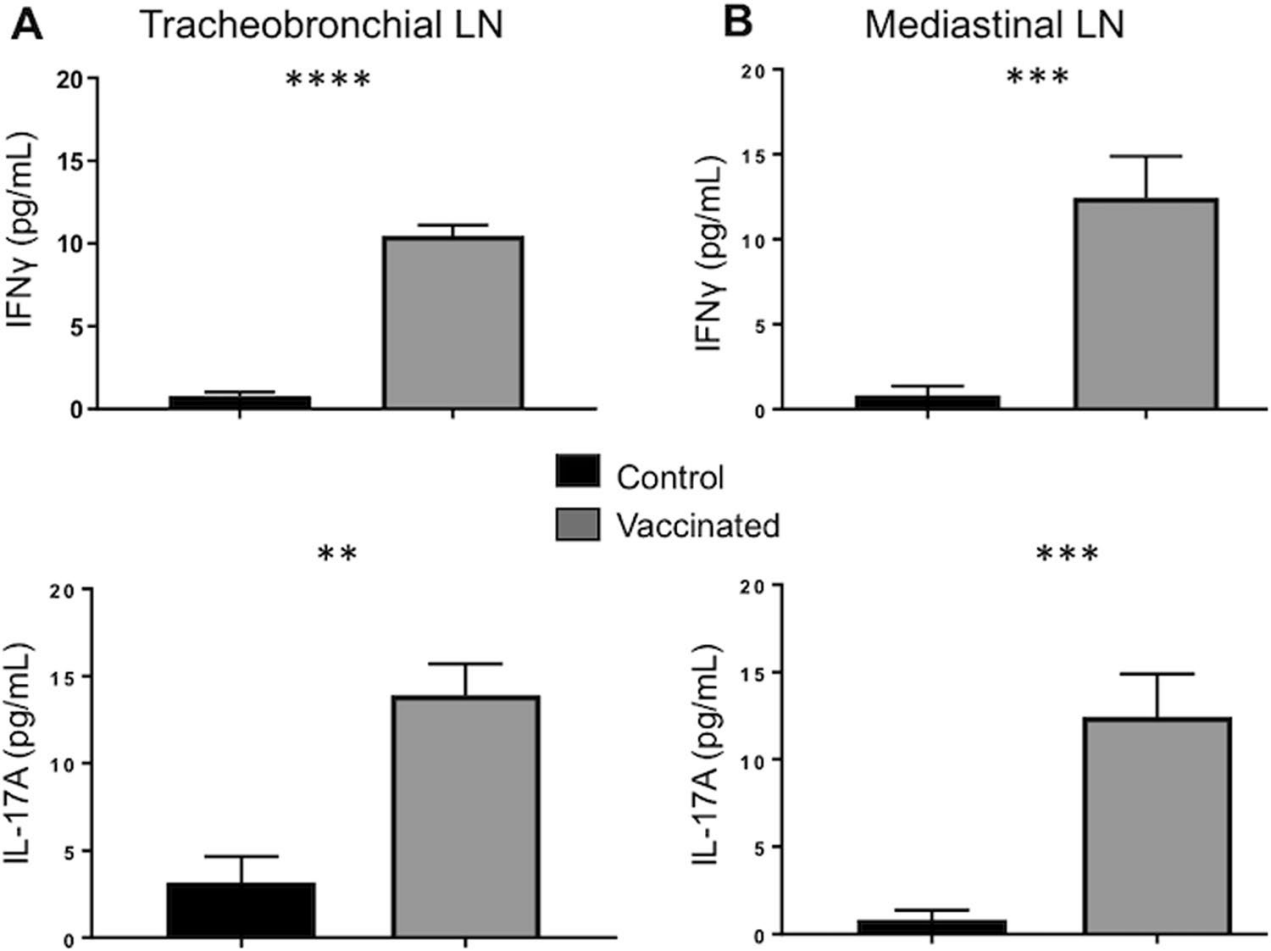
Cellular immune responses in lung-draining lymph nodes of BCG-vaccinated calves. Lung-draining tracheobronchial (**A**) and mediastinal lymph nodes (**B**), were collected from control (n = 7) or BCG–vaccinated animals (n = 7) during necropsy at 16 weeks after vaccination. Cells were isolated as described in Materials & Methods and restimulated for 6 days with PPD-b. Control wells remained unstimulated. Cell culture supernatants were collected from cultures and analyzed by commercial ELISA kit for IFNγ (upper panel) and IL-17 (lower panel). Data represent means ± SEM. **p < 0.01 ***p < 0.001 ****p < 0.001 as determined by Student’s t test. This figure was previously published^67^ and is duplicated with permission.

Vaccine-induced cellular immune responses were also evaluated in cells harvested from lung-draining lymph nodes during necropsy, 16 weeks after vaccination (Fig. 2). Single cell suspensions were prepared from the mediastinal and tracheobronchial LN, and *in vitro* antigen stimulation assays were performed as described in Materials & Methods. Cell culture supernatants were analyzed by sandwich ELISA for IFNγ (upper panels) and IL-17A (lower panels). Similar to the results from the BAL, cells isolated from the tracheobronchial (Fig. 2A) and mediastinal LNs (Fig. 2B) of BCG-vaccinated calves secreted IFNγ and IL-17A in specific response to *in vitro* PPD-b restimulation and the response was significantly increased over samples from unvaccinated controls at 16 week’s post vaccination.

### Aerosol BCG vaccination elicits effector and central memory T cell responses

*Ex vivo* assays are considered a measure of effector memory T cell responses. While most TB vaccines elicit *ex vivo* IFNγ responses, it is clear that not all vaccines that induce this response provide protection^2^. Long-term cultured IFNγ ELISPOT responses to TB vaccination negatively correlate with mycobacterial burden and TB-associated pathology and positively correlate with vaccine-induced protection^46–49^. In cattle and humans, the long-term cultured IFNγ ELISPOT assay evaluates central memory T cell responses following TB infection and vaccination^38,39^. Here, we determined if aerosol BCG vaccination was capable of eliciting both *ex vivo* and long-term cultured responses, as has been previously reported for other protective TB vaccines^39,46,47^.

**Figure 3:**
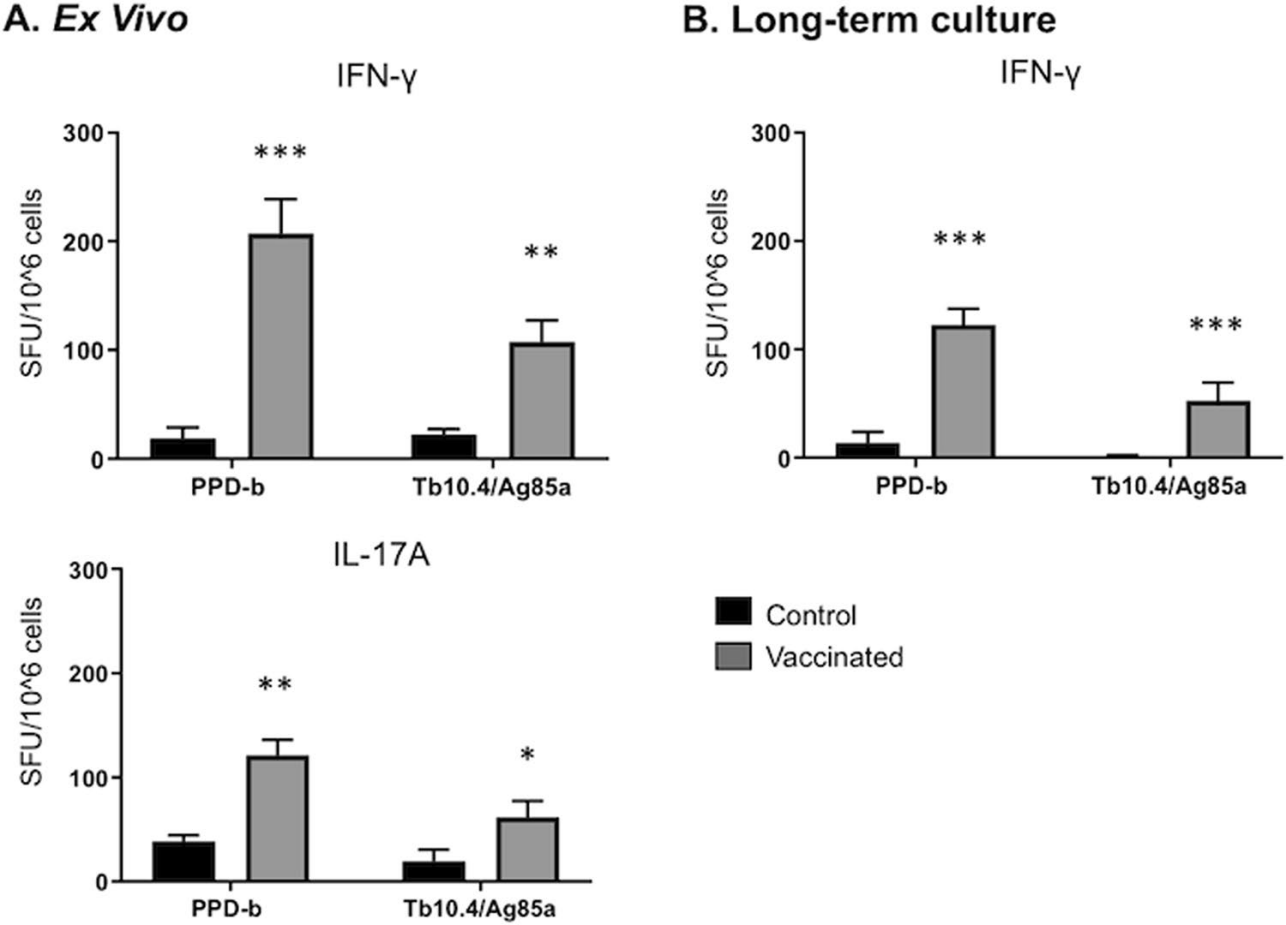
Long-term cultured and *ex vivo* IFNγ responses by cattle after *M. bovis* aerosol vaccination. Cultured ELISPOT analysis was performed ~8 weeks after BCG aerosol vaccination. (**A**) For the *ex vivo* response, freshly isolated PBMCs were stimulated with rTb10.4/Ag85a or PPD-b or medium alone for 20 h (IFNγ) or for 48 hours (IL-17A). (B) Long-term cultured cells were generated by stimulating PBMC with a cocktail of rTB10.4/rAg85A (1 μg/ml each) and PPD-b (200 IU/ml) for 13 days followed by transfer to ELISPOT plates with APCs and the addition of either rTb10.4/Ag85a, PPD-b or medium alone. Medium control responses were subtracted from antigen-stimulated responses and results are presented as mean spot forming cells (SFC)/10^6^ cells ( ± SEM) for (**A**) *ex vivo* conditions or (**B)** long-term culture *p < 0.05 **p < 0.01 ***p < 0.001 as determined by Student’s t test. This figure was previously published^67^ and is duplicated with permission.

PBMC were isolated and short term stimulations were performed to quantify the numbers of *ex vivo* effector IFNγ and IL-17A-secreting cells (spot-forming cells, SFC) by ELISPOT assays^39,50^. Aerosol BCG vaccination elicited significant numbers of effector PPD-b and rTb10.4/Ag85a-specific T cells in the peripheral blood (Fig. 3A**)**. Long-term cultures were performed to quantify the numbers of *M. bovis-*specific central memory T cells in peripheral blood. As seen in Fig. 3B, aerosol BCG vaccination elicited significant numbers of IFNγ-producing central memory T cells. Antigen-specific responses to rTb10.4/Ag85a and PPD-b both exceeded the long-term ELISPOT resposes measured from non-vaccinates (Fig. 3B). Thus, aerosol BCG vaccination of elicits strong recall effector and central memory responses in calves^51,52^.

### Aerosol BCG vaccination induces a robust systemic and mucosal antigen-specific γδ T cell responses

Having demonstrated that BCG vaccination via the respiratory tract elicited effector and memory T cell responses in the calf, we next investigated the differentiation status of the responding γδ T cell populations. Using flow cytometry, we assessed mycobacterial-specific γδ T cell responses in the peripheral and lung compartment. It is well established that aerosol BCG vaccination induces effector and memory CD4 T cell differentiation^53^; therefore, where relevant, we made comparisons to the *M. bovis-*specific CD4 T cell populations in the same animals.

**Figure 4:**
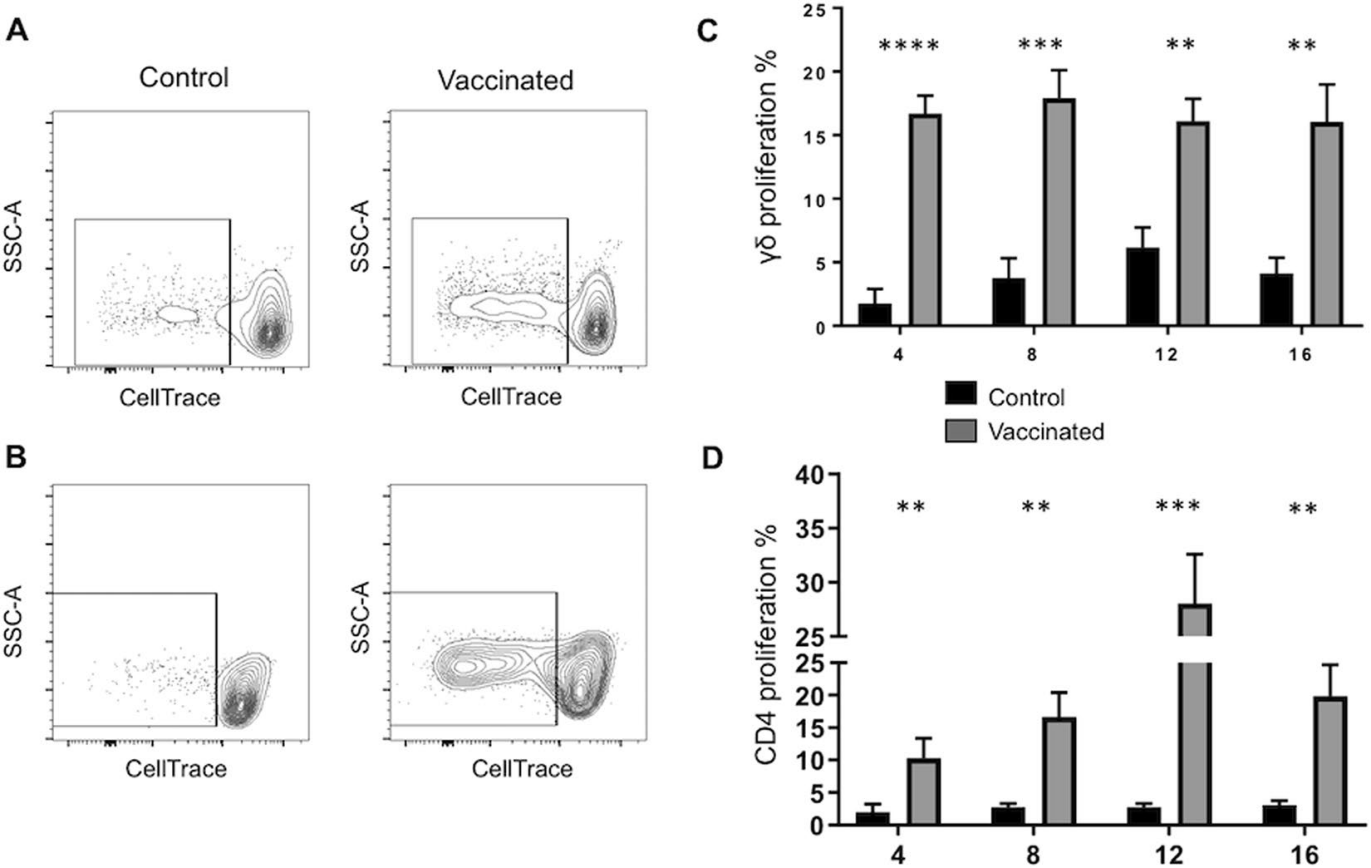
Systemic *M. bovis*-specific responses in response to *in vitro* restimulation with PPD-b. PBMCs from control (n = 10) or BCG–vaccinated animals (n = 18) were labeled with CellTrace, and 5 × 10^6^ cells/ml were cultured for 6 days in the presence or absence of PPD-b. Cells were labeled with anti-bovine γδ TCR or CD4 (see Table 1) and analyzed by flow cytometry for CellTrace dilution. (**A**,**B**) Representative contour plots of proliferative responses to *in vitro* PPD-b stimulation from a control and BCG-vaccinated animal. Gating hierarchy (gating sequence as depicted by the arrows): Single cells (SSC-A vs SSC-H), lymphocytes (SSC-A vs FSC-A), γδ T cells (**A**) or CD4 (**B**) and CellTrace dilution as shown in Fig. S1 (**C**,**D**) Cumulative percentage of γδ T cells (**C**) and CD4 T cells (**D**) from vaccinated and control animals that proliferated in response to PPD-b, as measured by CellTrace dilution, at 4-, 8- 12- or 16-weeks post vaccination. Analysis was performed with Flowjo software. Background proliferation was subtracted, and results represent change over unstimulated samples. Data represent means ± SEM. **p < 0.01 ***p < 0.001 ****p < 0.001 as determined by Student’s t test. This figure was previously published^67^ and is duplicated with permission.

Following 4-, 8-, 12- and 16-weeks post vaccination, PBMC were isolated, labeled with CellTrace dye and cultured for 6 days in the presence or absence of PPD-b. On day 6 of culture, cells were analyzed by flow cytometry for T cells that divided in response to PPD-b restimulation. Representative γδ and CD4 T cell flow plots from a vaccinated and control animal are depicted in Fig. 4A,B. Consistent with the results in Figs 1–3, aerosol BCG vaccination induced long-term, robust systemic cellular responses, and γδ T cells appear to participate in this response, as evidenced by the robust proliferative responses that persisted in vaccinated calves through at least 16-weeks post vaccination **(**Fig. 4C**)**. The observed γδ T cell recall responses were *M. bovis*-specific, as cells from non-vaccinated cattle did not respond to the complex mycobacterial antigens. Similarly, only CD4 T cells from BCG-vaccinated animals, and not control animals, participated in the recall response to the PPD-b antigen restimulation *in vitro* (Fig. 4B,D).

**Figure 5:**
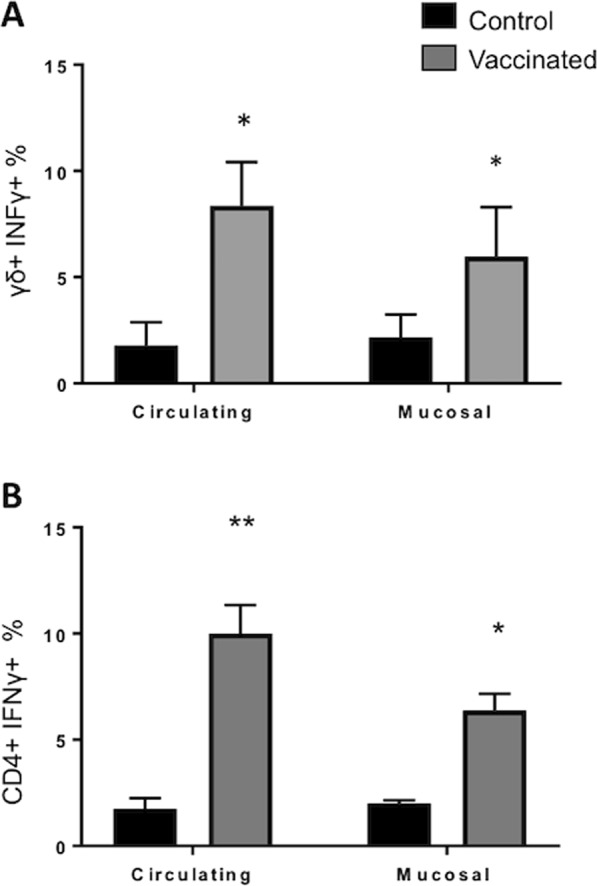
Circulating and airway *M. bovis*-specific IFNγ expression in response to *in vitro* restimulation with PPD-b. Approximately ~8 weeks after aerosol vaccination, IFNγ expression was analyzed in circulating (PBMC) and in local (BAL) compartment from control (n = 7) or BCG–vaccinated animals (n = 7). 1 × 10^6^ cells/well were stimulated *in vitro* with PPD-b (200 IU/ml) for 16 hours. Cells were then stained for intracellular IFNγ expression and analyzed by flow cytometry. Gating hierarchy (gating sequence as depicted by the arrows): Single cells (SSC-A vs SSC-H), lymphocytes (SSC-A vs FSC-A), γδ or CD4 T cells and IFNγ expression as shown in Figs S2 and S3 (**A**,**B**) The proportions of circulating and airway (**A**) γδ^+^ IFNγ^+^ and (**B**) CD4^+^ IFNγ^+^ conditions are shown. Analysis was performed with Flowjo software. Data represent means ± SEM. *p < 0.05, **p < 0.01 as determined by Student’s t test. This figure was previously published^67^ and is duplicated with permission.

The frequency of vaccine-elicited IFNγ-producing γδ T cells was determined by intracellular cytokine staining on PBMC and BAL cells after ~8 weeks post vaccination. Aerosol BCG vaccination induced *M. bovis-*specific, IFNγ-producing γδ T cells in both the peripheral and mucosal compartments (Fig. 5A). The frequency of γδ T cells producing IFNγ in response to PPD-b stimulation was significantly higher in animals that were vaccinated with BCG compared to unvaccinated controls. Similarly, aerosol vaccination induced IFN-γ-producing antigen-specific CD4 T cells in the peripheral and mucosal compartment (Fig. 5B). Thus, aerosol BCG vaccination induces functional, *M. bovis-*specific γδ T cells in both the lung and periphery.

### *M. bovis*-specific γδ T cells from BCG vaccinated calves adopt a memory phenotype based on CD27 and CD45R expression

We have recently shown that virulent *M. bovis* infection in cattle promotes the differentiation of circulating γδ T cells into subsets that share characteristics with human T_CM_ γδ T cells (CD45R- CD27 + CD62L^hi^)^41^. Here, we investigated the phenotype of both systemic and lung-associated γδ T cells to determine the impact of aerosol BCG vaccination on γδ T cell differentiation.

**Figure 6:**
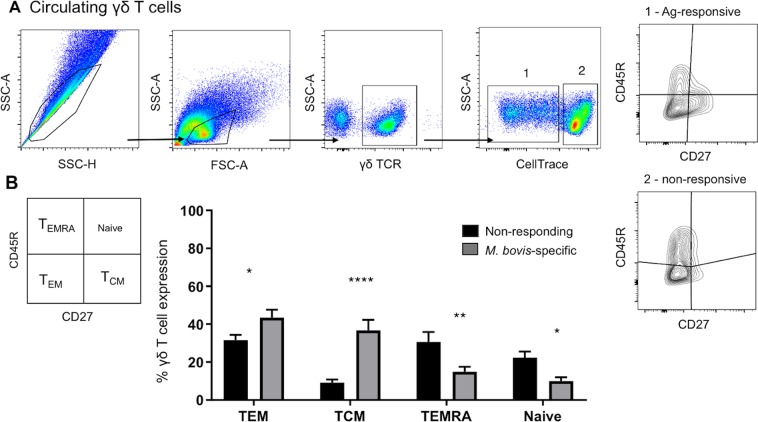
Phenotype of circulating antigen-specific γδ T cells. PBMCs were isolated from calves ~ 8 weeks after BCG vaccination. Cells were stained with CellTrace dye and incubated with *M. bovis* PPD-b for 6 days. Cells were analyzed by flow cytometry and the expression of CD45R and CD27 was evaluated on *M. bovis*-specific (proliferating) γδ T cells. (**A**) Gating hierarchy (gating sequence as depicted by the arrows): Single cells (SSC-A vs SSC-H), lymphocytes (SSC-A vs FSC-A), γδ T cells, proliferating (1) and non-proliferating cells (2) in response to PPD-b and analyzed for CD27 versus CD45R expression. (**B**) Proportion of *M. bovis-*specific (CellTrace dim; grey bars) and nonspecific (CellTrace bright; black bars) γδ T cells in CD45R/CD27 defined subsets (Naïve: CD45R^+^CD27^+^; T_CM_: CD45R^−^CD27^+^; T_EM_: CD45R^−^CD27^−^; T_EMRA_: CD45R^+^CD27^−^). Data are presented as ± SEM. *p < 0.05; **p < 0.01, ****p < 0.0001 indicates a significant difference from antigen-responsive cells compared to non-responsive cells as determined by Student’s t-test. This figure was previously published^67^ and is duplicated with permission.

In the circulating compartment, aerosol BCG vaccination induced a significant increase in γδ T cells with a T_CM_ (CD45R^−^ CD27^+^) phenotype, and a moderate increase in γδ T cells with a T_EM_ (CD45R^−^ CD27^+^) phenotype (Fig. 6A). The cumulative proportions of effector/memory γδ T cells from all calves are shown in Fig. 6B. Our results suggest that BCG vaccination, similar to virulent *M. bovis* infection, elicits a γδ T_CM_ phenotype in the circulation that comprises the majority of the *M. bovis*-specific proliferative response. Interestingly, however, in contrast to our previous studies with virulent *M. bovis* infection, BCG vaccination also induced the expansion of circulating γδ T cells with a T_EM_ (CD45R^−^ CD27^+^) phenotype.

**Figure 7:**
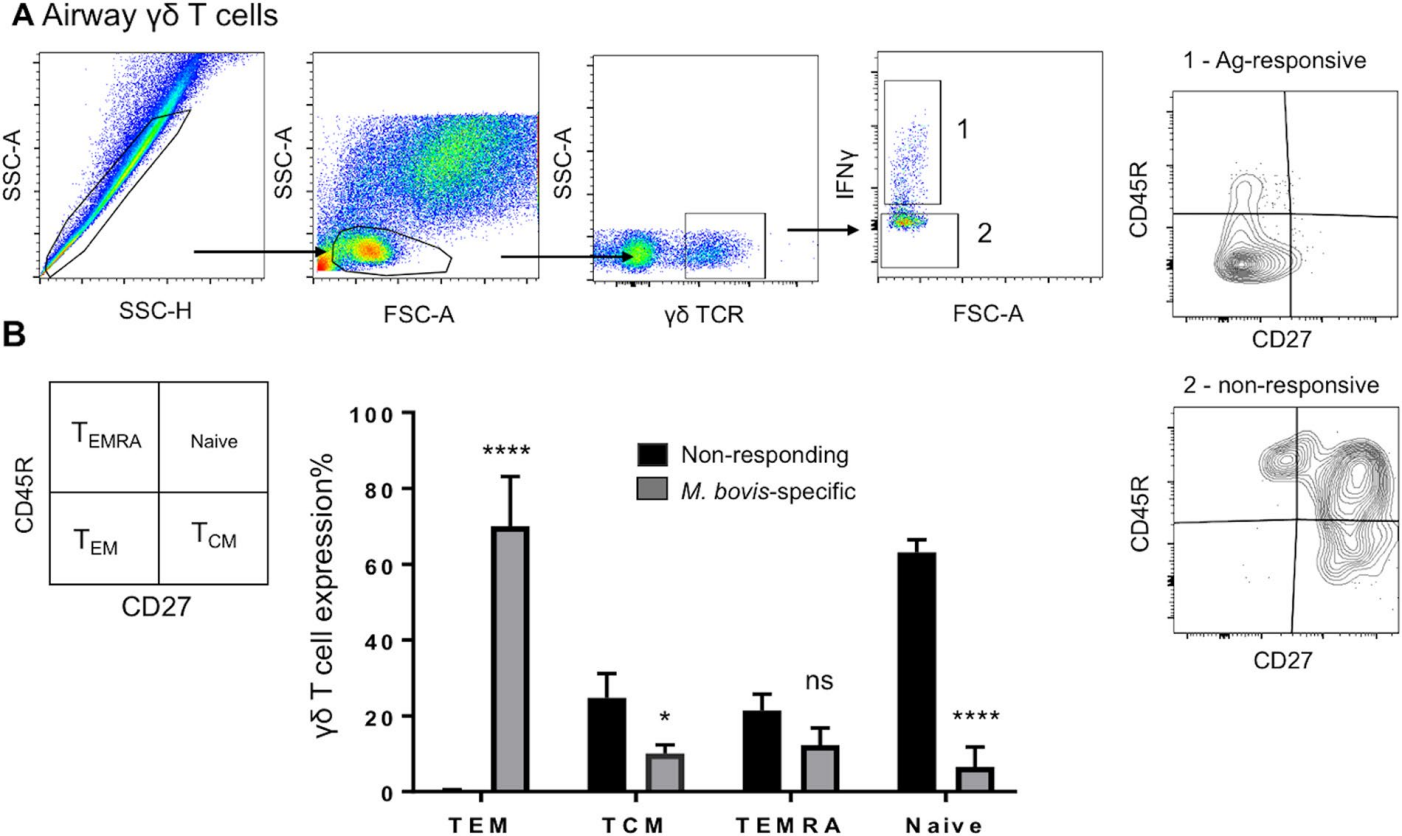
Phenotype of airway antigen-specific γδ T cells. BAL cells were isolated from control (n = 7) or BCG–vaccinated (n = 7) calves ~8 weeks after vaccination. Cells were stimulated with PPD-b *in vitro* for 16 hours. Cells were surface stained and stained for intracellular IFNγ, and then analyzed by flow cytometry to evaluate CD45R and CD27 expression on *M. bovis*-specific γδ T cells. (**A**) Gating hierarchy (gating sequence as depicted by the arrows): Single cells (SSC-A vs SSC-H), lymphocytes (SSC-A vs FSC-A), γδ T cells, IFNγ^+^ (grey bars) and IFNγ^neg^ (black bars) cells and CD27 versus CD45R expression. (**B**) Proportion of *M. bovis-*specific (IFNγ^+^; grey bars) and nonspecific (IFNγ^neg^; black bars) γδ T cells in CD45R/CD27 defined subsets (Naïve: CD45R^+^CD27^+^; T_CM_: CD45R^−^CD27^+^; T_EM_: CD45R^−^CD27^−^; T_EMRA_: CD45R^+^CD27^−^). Data are presented as ± SEM. *p < 0.05, ****p < 0.0001 indicates a significant difference from antigen-responsive cells compared to non-responsive cells as determined by Student’s t-test. This figure was previously published^67^ and is duplicated with permission.

In the mucosal compartment, we observed that aerosol BCG vaccination preferentially induced the differentiation of γδ T_EM_ (CD45R^−^ CD27^+^) cells, with low proportions of T_CM_ cells compared to the circulating population (Fig. 7A**)**. The relative contribution of lung γδ T cell subsets in the response to PPD-b are shown in Fig. 7B.

Thus, BCG vaccination via the respiratory route induces the differentiation of antigen-specific γδ T cells with a T_CM_ phenotype in the periphery, and a T_EM_ phenotype in the airways.

### *M. bovis*-specific γδ T cells modulate expression of the tissue-associated chemokine receptors CXCR3 and CCR5

Expression of the chemokine receptors CXCR3 and CCR5 has been used to differentiate effector and memory T cell subsets (35). We have previously shown that circulating γδ T cells modulate their expression of CXCR3 and CCR5 during virulent *M. bovis* infection^41^. The increased expression of CCR5 and CXCR3 on *M. bovis*-responsive T cells is expected to enable these populations to migrate into the inflamed tissue. Therefore, we next investigated the effect of aerosol BCG vaccination on the acquisition of lung homing receptors by *M. bovis* responsive γδ T cell in peripheral blood and BAL cells.

**Figure 8:**
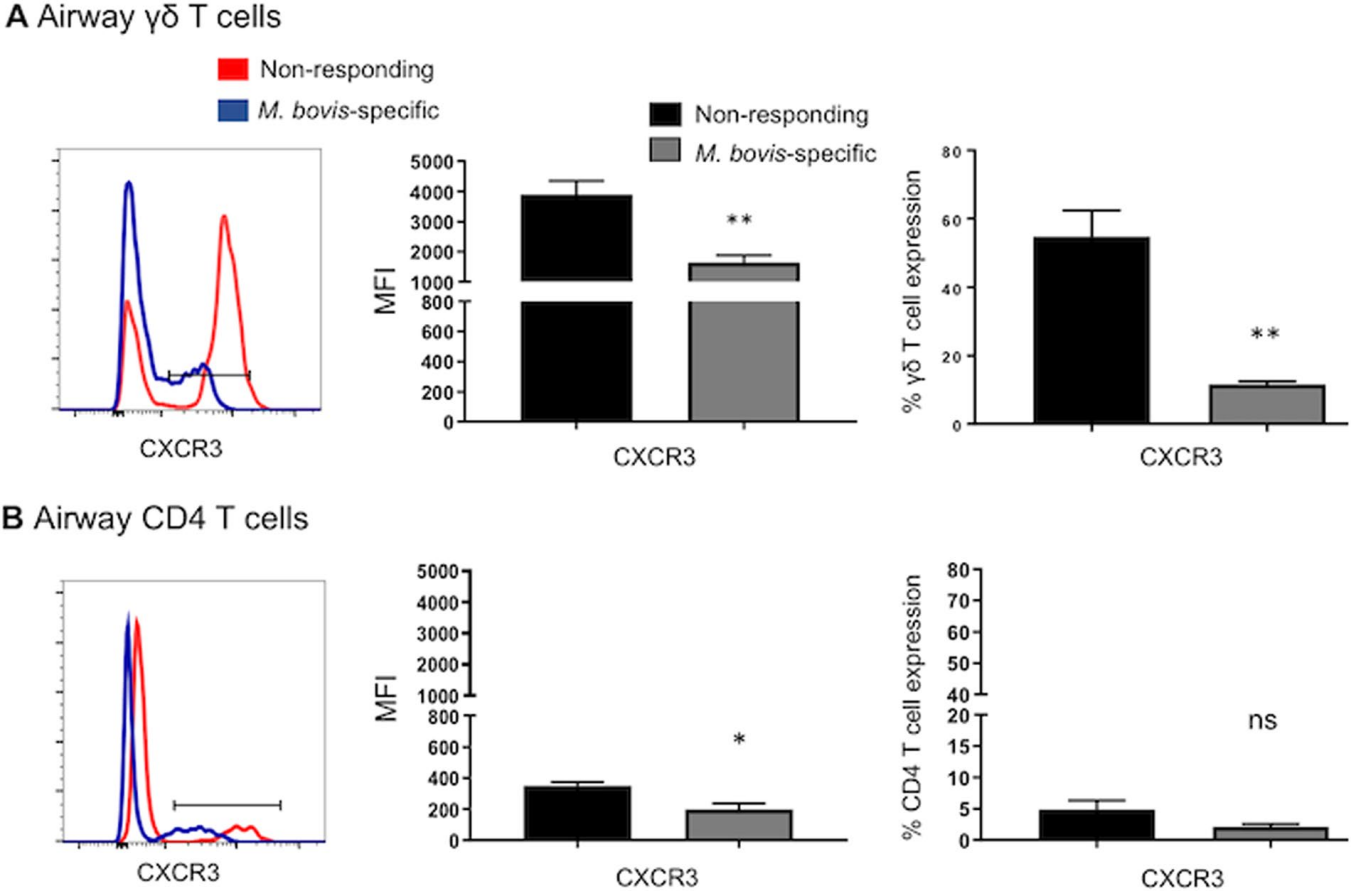
Surface expression of chemokine receptors on mucosal *M. bovis*-specific γδ and CD4 T cells. BAL cells were isolated from control (n = 7) or BCG–vaccinated (n = 7) calves ~8 weeks after vaccination. Cells were stimulated with PPD-b *in vitro* for 16 hours. Cells were surface stained, stained for intracellular IFNγ expression, and then analyzed by flow cytometry for expression of CXCR3. Gating hierarchy (gating sequence as depicted by the arrows): Lymphocytes (SSC-A vs FSC-A), γδ T cells or CD4 cells, IFNγ^+^ and IFNγ^neg^ cells and CXCR3 as shown in Fig. S4. The left panels depict representative histogram plots of CXCR3 expression on *M. bovis*-specific γδ (**A**) and CD4 T cells (**B**). Cumulative results from airway γδ T cells (**A)** and CD4 T cells (**B**) are depicted: relative expression (MFI) (middle panels) and the frequency of CXCR3^+^ cells (right panels) on *M. bovis*-specific (grey bars) or *M. bovis* nonresponsive (black bars). Data are presented as mean ± SEM. Not significant (ns), *p < 0.05, **p < 0.01 indicates a significant difference from antigen-responsive cells compared to non-responsive cells as determined by Student’s t-test. This figure was previously published^67^ and is duplicated with permission.

**Figure 9:**
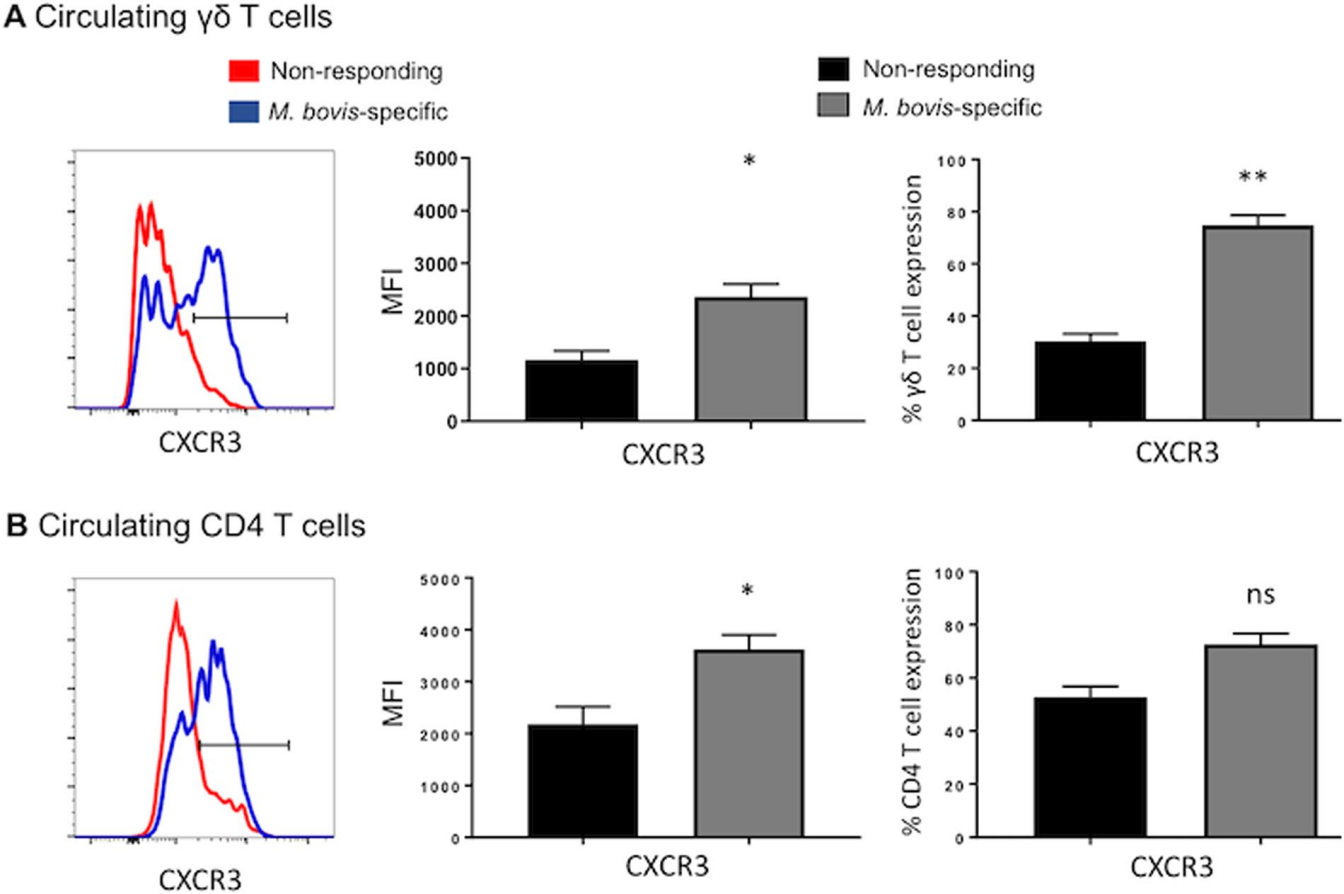
Surface expression of chemokine receptors on circulating *M. bovis*-specific γδ and CD4 T cells. (**A**,**B**) PBMCs were isolated from calves ~8 weeks after vaccination. Cells were stained with CellTrace dye and incubated with PPD-b for 6 days. Cells were surface stained and then analyzed by flow cytometry to study CXCR3 and CCR5 expression on circulating *M. bovis*-specific γδ (**A**) and CD4 T cells (**B**). Gating hierarchy (gating sequence as depicted by the arrows): Lymphocytes (SSC-A vs FSC-A), γδ T cells or CD4 cells, proliferating and non-proliferating cells (CellTrace dilution) and CXCR3 or CCR5 expression as shown in Fig. S5. The left panels depict representative histogram plots of CXCR3 expression on *M. bovis*-specific γδ (**A**) and CD4 T cells (**B**). Cumulative results from blood γδ T cells (**A)** and CD4 T cells (**B**) are depicted: relative expression (MFI) (middle panels) and the frequency of CXCR3^+^ cells (right panels) on *M. bovis*-specific (grey bars) or *M. bovis* nonresponsive (black bars). Data are presented as mean ± SEM. Not significant (ns), *p < 0.05, **p < 0.01 indicates a significant difference from antigen-responsive cells compared to non-responsive cells as determined by Student’s t-test. This figure was previously published^67^ and is duplicated with permission.

We observed marked differences in the patterns of chemokine receptor expression by *M. bovis*-responsive γδ T cells isolated from mucosal versus peripheral compartments. The majority of *M. bovis*-specific γδ T cells recovered from the BAL expressed lower surface levels of CXCR3 compared to non-responding γδ T cells (Fig. 8A**)**. As seen in Fig. 8B, airway antigen-specific CD4 T cells also expressed lower levels of CXCR3 compared to nonresponsive CD4 T cells. Comparison of chemokine receptor expression by mucosal γδ T cells compared with circulating *M. bovis-*specific γδ T cells, revealed significantly higher expression of CXCR3 by circulating antigen-specific populations (Fig. 9C**)**. The expression of CCR5 on both CD4 and γδ T cells followed a similar trend, with increased expression by circulating, *M. bovis-*specific cells and reduced by *M. bovis-*responsive T cells reovered from the airways; however, this change was not statistically significant (Supp. Fig. 6). Together our data show that aerosol BCG vaccination induces up-regulation of CXCR3 by circulating γδ T cells, which may confer lung homing properties under inflammatory conditions and correlate with the observed effector memory phenotype (Fig. 7)^36^. Conversely, analysis of airway, *M. bovis-*responsive γδ T cells indicates a tendency towards downregulation of chemokine receptor expression, perhaps indicating that T cells localized to the lung no longer require these receptors.

## Discussion

Current vaccine regimens against TB are ineffective; thus, development of improved intervention strategies and a better understanding of the nature of protective immunity remain important goals for TB research. Vaccination via the respiratory tract has been demonstrated to induce significant protection against virulent mycobacterial challenge^4–9^. Although Th1 cells are of major importance for immunity to TB, other immune populations, notably γδ T cells [^9,31,54,55^ and reviewed^24^], may represent another essential component for the anti-mycobacterial immune response. The focus of this study was to investigate the immunogenicity, and generation of systemic and local γδ T cell responses to BCG vaccination via the respiratory tract.

Infants are an important target population for TB vaccination. However, the infant immune system represents significant challenges when designing vaccines [reviewed^56^]. There are few suitable models suitable for studying infant immunology to TB, and most mucosal TB vaccines have been studied in adult animals. Although we often attempt to extrapolate our knowledge of host defense and vaccine-induces immunity from adults to infants, we currently know little about neonatal immune response to mucosal vaccination. Furthermore, despite the promise of a vaccine strategy, there are safety concerns associated to the administration of a live replicating pathogen directly to the respiratory mucosa of immunosuppressed individuals. Thus, a reliable animal model that reflects the immune response to this route of vaccination in human infants is urgently required. Here, we observed that aerosol BCG immunization of young calves is highly immunogenic and induces antigen-specific immune responses in both the systemic and local compartment (Figs 1 and 2). Our results are consistent with those reported by Hoft *et al*., showing that oral delivery of BCG to humans induced both systemic and mucosa-associated immune responses in the airways^57^. The capacity of aerosol BCG vaccination to induce responses in both the systemic and local immune compartments in cattle is in contrast to the observations made in mice, where vaccine responses are compartmentalized following mucosal vaccination^58^. In non-human primates, responses are less compartmentalized, but the magnitude of response correlates with the route of vaccination, with strong systemic responses after systemic vaccination and strong responses in the respiratory tract in animals immunized by aerosol delivery^5^. Given the limitations of other animal models, our results are encouraging and demonstrate the potential of the calf model to evaluate the safety and immunogenicity of other TB vaccine candidates through this route of immunization.

In parallel with efforts to evaluate the immunogenicity of the aerosol route of delivery, the identification of immunological correlates of protection is essential to reduce costs of challenge experiments and to prioritize vaccine candidates. Assessing the magnitude and frequency of IFNγ-producing CD4 T cells has remained the standard measurement of vaccine induced T cell memory for many years. However, it has become clear recently that this ex *vivo* assay cannot consistently predict vaccine success^59^. Alternatively, long-term cultured IFNγ ELISPOT responses to TB vaccination have been shown to negatively correlate with mycobacterial burden and TB-associated pathology, and to positively correlate with vaccine success^46–48,50,60^. In humans and cattle, the cultured ELISPOT assay is a surrogate of T_CM_ responses^39,46,47^. Using this assay and the conventional ELISPOT, we evaluated if aerosol BCG vaccination was capable of eliciting both effector and T_CM_ responses. Consistent with the results of previous bovine TB vaccine trials examining parenteral BCG vaccination^46,48^, we demonstrate here that aerosol BCG vaccination elicits significant long-term cultured IFNγ ELISPOT responses to both PPD-b and the protein antigens Tb10.4 and Ag85a (Fig. 3). Thus aerosol BCG vaccination elicits effector and central memory T cell (T_CM_) responses in both humans and cattle^39,48,51,52^.

In humans, serious TB infection induces expansion of phenotypically immature, T_CM_ Vγ9Vδ2 T cells, and a reduction in the pool of Vγ9Vδ2 T cells with immediate effector functions (T_EM_ and T_EMRA_ cells), suggesting a functional impairment of γδ T cells perhaps, due to a persistent stimulation^61^. Similarly, we have recently reported that virulent *M. bovis* infection in cattle can significantly alter the differentiation of circulating *M. bovis*-specific γδ T cells towards a T_CM_ (CD45R- CD27 + CD62L^hi^) phenotype^41^. In this study, phenotypic analyses of circulating γδ T cells showed that *M. bovis-*specific γδ T cells are strongly affected by BCG vaccination; however, in contrast to virulent infection, the vaccine strain induced the expansion of both T_CM_ and T_EM_ γδ T cells in the blood (Fig. 6). As particular memory subsets of CD4 T cells have been shown to correlate with reduced mycobacterial burden, or to indicate disease progression, we speculate that changes in the differentiation status of memory γδ T cell subsets may also be indicative of particular disease states or serve as a correlate of immune status.

Previous reports in humans have shown that T_EM_ γδ T cells are highly represented in sites of inflammation and display immediate effector functions^33^. Consistent with these findings, BCG vaccination via the respiratory tract was associated with an increase in airway IFNγ^+^ γδ T cells (Fig. 5) which exhibited a T_EM_ phenotype (Fig. 7). In non-human primates, accumulation of phosphoantigen-specific Vγ2Vδ2 T cells in the lungs significantly reduces *M. tb* burden and associated lung pathology^9,28^. Similarly, in mice, *M. bovis* BCG intranasal infection elicits increased levels of IFNγ^+^ γδ T cells with cytotoxic activity against infected macrophages^42^. Thus, accumulation of T_EM_ *M. bovis*-responsive γδ T cells in the airway mucosa may enable an immediate response upon pathogen re-exposure. Identifying strategies to elicit memory γδ T cell differentiation with a high frequency of γδ T cell effectors in the lung, as well as peripheral mycobacteria-specific γδ T cells that have the capacity to rapidly traffic into the lung to enhance and sustain protection, may be expected to confer optimal vaccine-induced protection^38,39^

Few studies have examined γδ T cell chemokine expression^33^; however, mycobacterial antigen stimulation appears to alter γδ T cell chemokine receptor expression^37^. In our previous studies, we have observed increased expression of both CXCR3 and CCR5 on circulating γδ T cells from cattle infected with virulent *M. bovis*^41^. Similarly, we show here that aerosol BCG vaccination is associated with an increase in surface expression of CXCR3 in circulating *M. bovis*-specific γδ T cells compared to non-responding cells (Fig. 9). CXCL10, the ligand of CXCR3, is involved in trafficking of Th1 lymphocytes to areas of inflammation. It has been shown that peripheral human Vγ2Vδ2 CXCR3^+^ T cells are recruited efficiently by CXCL10^37,62^. Moreover, CXCL10 is found *in vivo* in lymph nodes and TB granulomas^63^, suggesting that CXCR3 expression in circulating γδ T cells may enable rapid migration to the lungs upon *M. bovis* challenge^64,65^. Interestingly, comparison of CXCR3 expression on circulating T cell populations with *M. bovis-*specific T cells recovered from the BAL on the same day, revealed significantly reduced surface expression by both γδ and CD4 T cells in the airways (Fig. 9A,B). These findings are intriguing, as Poggi *et al*. showed that the homeostatic chemokine CCL21 was more efficient at inducing transendothelial migration of lung-resident Vγ2Vδ1 CXCR3^+^ T cell populations than inflammatory CXCL10, suggesting that lung-associated γδ T cells preferentially respond to homeostatic chemokines, while circulating Vγ2Vδ2 CXCR3^+^ T cells are more sensitive to inflammatory chemokines and thus might not require high expression of CXCR3^62^. It may also be possible that CXCR3 is internalized or shed by cells which have migrated to the lung, thus explaining the reduced surface expression. Functional studies to assess CXCR3-dependent lymphocyte trafficking were not performed in this study, thus its importance in facilitating γδ T cell recruitment during *M. bovis* infection in the bovine remains to be fully determined.

BCG remains the most efficacious, ‘gold-standard’ TB vaccine available to prevent TB in humans and animals. Therefore, until improved vaccines are available, it is crucial to identify the most effective strategies for using BCG. As observed here, mucosal vaccination in juvenile calves is immunogenic, priming robust effector and memory *Mycobacterium*-specific T cell responses in both peripheral and lung compartment. Based on our observations, there is merit in evaluating this route of immunization using other vaccine candidates, and in employing the infant calf model, as it can provide critical insights into local TB-specific immune responses in infant humans. Through a comparative assessment of the phenotype and functions of *M. bovis*-specific γδ T cells in the systemic and mucosal compartment, we have shown that a single BCG immunization via the respiratory tract induces differentiation of T_CM_ and T_EM_ *M. bovis-*responsive γδ T cells, with a predominance of T_EM_ γδ T cells in the lungs, where they would be expected to mediate immediate effector functions and rapid protection against TB invasion. γδ T cells are known to promote resistance to *Mycobacterium* infection^9,10,32^. Thus, engaging γδ T cells through novel, aerosol vaccination strategies may be particularly beneficial for promoting local protection in the respiratory tract.

## Materials and Methods

### Animal use ethics

All animal procedures were conducted in strict accordance with federal and institutional guidelines and were approved by the Kansas State University Institutional Animal Care and Use Committee (Protocol Number: 27–2956). A total of 28 Holstein steers (~6-8-week-old) were used in the following experiments. Animals were housed in outdoor pens at the College of Veterinary Medicine, Kansas State University in Manhattan, KS. Animals had ab libitum access to hay, water, and concentrate. Steps were taken to avoid prolonged restraint and discomfort during all handling procedures. Body temperature was assessed if animal demonstrated signs of clinical illness and antibiotics and analgesics were administered as needed if animals presented with clinical disease independent of the experimental protocol. At the end of the study animals were humanely euthanized by barbiturate overdose. The calves showed no clinical signs following administration of the vaccine, and we observed no pathological changes in the lungs or tissues of the respiratory tract at euthanasia.

### *Mycobacterium bovis* BCG aerosol vaccine procedures

*M. bovis* BCG Danish strain was a gift from Dr. Ray Waters at the National Animal Disease Center, USDA. BCG was prepared using standard techniques in Middlebrook 7H9 liquid media (Becton Dickinson, Franklin Lakes, NJ) supplemented with 10% oleic acid-albumin-dextrose complex (OADC) plus 0.05% Tween 80 (Sigma, St. Louis, Missouri). For the first experiment, treatment groups consisted of non-vaccinated steers (n = 3) and animals receiving 1 × 10^8^ colony-forming units (CFU) of *M. bovis* BCG Danish strain (n = 11). For the second study, treatment groups consisted of non-vaccinated calves (n = 7) and animals receiving 1 × 10^8^ CFU of *M. bovis* BCG Danish strain (n = 7). For both studies, BCG inoculum was delivered to restrained calves by aerosol as described by Palmer *et al*.^66^. Briefly, inoculum was nebulized into a mask (Trudell Medical International, London, ON, Canada) covering the nostrils and mouth, allowing regular breathing and delivery of the bacterial inoculum to the respiratory tract. Clinical signs, including cough, dyspnea, and loss of appetite were monitored daily throughout the study. No clinical signs or pathology associated with BCG immunization were observed.

### PBMC isolation

Peripheral blood was drawn from the jugular vein into 2 × acid-citrate-dextrose solution. For peripheral blood mononuclear cells (PBMC) isolation, blood was diluted 1:1 in phosphate-buffered saline (PBS), cells were collected from buffy coat fractions, and PBMCs were isolated by density centrifugation on Histopaque (Sigma). Contaminating red blood cells were removed using RBC lysis buffer. Finally, cells were washed three times, counted in an hemocytometer and resuspended in complete RPMI (cRPMI) composed of RPMI-1640 (Gibco, Carlsbad, CA) supplemented with 2 mM L-glutamine, 25 mM HEPES buffer, 1% antibiotic-antimycotic solution, 1% non-essential amino acids 2% essential amino acids, 1% sodium pyruvate, 50 μM 2-mercaptoethanol (all from Sigma, St. Louis, MO), and 10% (v/v) heat-inactivated fetal bovine sera (FBS).

### Bronchoalveolar lavage fluid collection and cell isolation

Broncheoalveolar lavage (BAL) samples were collected using a modified stallion urinary catheter (JorVet, Jorgensen Laboratories). The catheter was blindly passed through the nose and advanced through the trachea until lodging in the bronchus. A total of 180 mL of sterile saline was divided into three aliquots. An aliquot was first introduced to the lower respiratory tract, followed by immediate suction to obtain lower airway washes. The procedure was repeated twice more. All three aliquots were pooled at the end of the procedure. BAL samples were kept on ice, filtered over sterile gauze, and centrifuged at 200 × g for 10 minutes at 4 °C. Contaminating red blood cells were removed using RBC lysis buffer. Cells were washed, resuspended in cRPMI, counted and 1 × 10^6^ cells/well were resuspended in cRPMI and plated in 96 well plates. Subsequently, BAL cells were cultured for 6-days at 37 °C, in the presence of 5% CO2, and stimulated with M. bovis PPD (PPD-b, 200 IU/mL, Prionics Ag, Sclieren, Switzerland). Concanavalin A mitogen (ConA, 5 µg/ml, Sigma) or cRPMI medium were used as positive and negative control, respectively. Plates were incubated for 72 hours for cytokine measurement at 37 °C, 5% CO_2_ incubator.

### Tissue collection and cell suspension

All calves were euthanized 16 weeks after vaccination by intravenous administration of barbiturate overdose. Tissues were examined for gross lesions. Mediastinal and tracheobronchial lymph node tissues were collected and placed in cold cRPMI. Cells from lymph nodes were gained by sieving small pieces of tissue through steel meshes. Contaminating red blood cells were removed using RBC lysis buffer. Finally, cells were washed three times, counted in a hemocytometer and resuspended in cRPMI. Subsequently, cells (5 × 10^5^/well) were plated in round-bottom 96-well plate in duplicates, cultured for 6-days at 37 °C, in the presence of 5% CO_2_, and stimulated with *M. bovis* PPD (PPD-b, 200 IU/mL, Prionics Ag, Sclieren, Switzerland),Concanavalin A mitogen (ConA, 5 µg/ml, Sigma) or cRPMI medium were used as positive and negative control, respectively.

### Long-term cell culture and ELISPOT

Following PBMC isolation, long-term cell culture was performed as described by Maggioli *et al*.^39,50^. Briefly, PBMC were cultured (2 × 10^6^ cells/well) in 24 well flat-bottom microtiter plates (Nunc, Thermo Fisher, Waltham, MA) and stimulated with a cocktail of *M. bovis* PPD (PPD-b, 200 IU/ml, Prionics Ag, Sclieren, Switzerland), rTB10.4 and rAg85A (1 μg/ml each) in cRPMI medium for 12 days 37 °C, 5% CO_2_. Media containing human rIL-2 (Sigma, 10 IU/ml) was used to replace media from the PBMC cultures at days 3 and 7. Fresh media without IL-2 was used at days 10 and 12. At day 13, autologous APCs were isolated by adherence incubating 2 × 10^5^/well of freshly isolated PBMC in complete medium at 37 °C, 5% CO_2_ for 90 min in 96-well ELISPOT plates (Millipore, Watford, UK) previously coated overnight with anti-bovine IFNγ capture-mAb (Kingfisher) and blocked in cRPMI, for 2 h at 37 °C, 5% CO_2_. Non-adherent cells were discarded, and the adherent cells washed twice times with warm cRPMI. Long-term cell cultures and antigen cocktail were added (2 × 10^4^/well) to the ELISPOT plate and incubated for 20 h at 37 °C, 5% CO_2_ in the presence of autologous APC.

### *Ex vivo* ELISPOT

The IFNγ and IL-17A ELISPOT assay was performed as described by Maggioli *et al*.^39,50^. Briefly, 96-well ELISPOT plates (Millipore) were coated at 4 °C overnight with an anti-bovine IFNγ capture mAb or IL-17A capture mAb (both from Kingfisher Biotech, Inc., St. Paul, MN), followed by a blocking step in cRPMI, for 2 h at 37 °C, 5% CO_2_. Fresh isolated PBMC or long-term cultured cells (2 × 10^4^/well) were added to ELISPOT plates and stimulated with either PPD-b (5 μg/ml), rTB10.4 and rAg85A (1 μg/ml each), ConA (5 µg/ml) or medium alone. Plates were incubated 20 h (IFNγ) or 48 h (IL-17A) at 37 °C, 5% CO_2_. Spot forming cells (SFC) were detected following the Vectastain ABC-AP Kit (Vector Laboratories, Burlingame, CA) standard procedures.

### Proliferation

Isolated PBMC were labeled using CellTrace Violet (Invitrogen, Carlsbad, CA) prior to cell culture following manufacturer’s instructions. Briefly, freshy isolated PBMCs were resuspended at 1 × 10^7^ cells/mL in PBS containing 10 μM/ml of the CellTrace dye. After gently mixing, PBMCs were incubated for 20 min at 37 °C in a water-bath. Labeling was quenched by using an equal volume of FBS, and cells were washed three times with RPMI medium. Subsequently, cells (5 × 10^5^/well) were plated in round-bottom 96-well plate in duplicates, cultured for 6-days at 37 °C, 5% CO_2_, in the presence of *M. bovis* PPD (PPD-b, 200 IU/mL). ConA (5 µg/ml) or cRPMI medium were used as positive and negative control, respectively. After six days, cells were surface stained (see Flow cytometry section) and analyzed for proliferation and surface marker expression by flow cytometry.

### Flow cytometry

Following the appropriate culture duration, cells were stained with primary and secondary monoclonal antibodies (mAbs) listed on Table 1. All incubation steps for staining were performed in FACS buffer (PBS with 10% FBS and 0.02% NA-azide) and incubated for 25 min at 4 °C. Cells were washed and fixed with BD FACS lysis buffer (BD Biosciences, Mountain View, CA) for 10 min at room temperature, washed and resuspended in FACS buffer until analysis.

**Table 1:** Flow cytometry reagents.

Reagent or antibody clone	Specificity, Source	Secondary antibodies, Source
ILA11	Bovine CD4, Washington State University	Allophycocyanin
GB21A	Bovine TCR1 delta chain, Washington State University	APC-Cy7, SouthernBiotech
GC6A	Bovine CD45R, Washington State University	PercpCy5.5, Life technologies
M-T271	Human CD27, Biolegend	Allophycocyanin or Pe-Cy7, Life technologies
G025H7	Human CXCR3 FITC, Biolegend	Not applicable
HM-CCR5	Human CCR5 PerCP/Cy5.5, Biolegend	Not applicable
CC302	Bovine IFN-γ-PE, Serotec (BioRad)	Not applicable
Live Dead Aqua	Dead cells, Invitrogen	Not applicable
CellTrace Violet	Not applicable, Life technologies	Not applicable

For intracellular staining, 1 × 10^6^ cells were incubated in cRPMI containing PPD-b for 16 h at 37 °C, 5% CO_2_ with Brefeldin A (GolgiPlug; BD Pharmingen, 10 μg/ml) added at 4 h of culture. After staining cell-surface markers, cells were permeabilized for 30 min using BD CytoFix/CytoPerm solution (BD Biosciences) and incubated with IFNγ (Table 1) for 45 min. Non-stimulated samples served as negative controls. Samples were acquired using a BD LSRII Fortessa flow cytometer (BD Biosciences). Data were analyzed using Flowjo software (Tree Star Inc., San Carlos, CA). Lymphocytes were identified in PBMC and BAL samples as shown in Supplemental Figs and were further subdivided by their CD4 and γδ T cell expression patterns.

### ELISA

PBMC and BAL cells were stimulated *in vitro* with PPD-b as described above. Plates were incubated for 6 days (PBMC) or 72 hours (BAL) at 37 °C, 5% CO_2_ incubator. Cell culture supernatants were stored at −80 °C until ELISA analysis. The concentration of cytokines in cell culture supernatants was determined using bovine commercial ELISA kits for IFNγ, and IL-17A (all from Kingfisher) according to manufacturer’s instructions. Each sample assayed was measured in duplicate.

## Statistical analyses

Results are expressed as averages ± standard errors of the mean (SEM). Statistical significance was determined by one-way Analysis of Variance (ANOVA) followed by Bonferroni test, or Student’s t test using Prism software (GraphPad, La Jolla, CA).

## Supplementary information

Supplemental Figures

## Data Availability

The datasets generated during and/or analysed during the current study are available from the corresponding author on reasonable request.

## References

[1] World Health Organization. Global tuberculosis report 2018. 231 (2018).

[2] Waters WR, Palmer MV, Buddle BM, Vordermeier HM (2012). Bovine tuberculosis vaccine research: historical perspectives and recent advances. Vaccine.

[3] Butkeviciute E, Jones CE, Smith SG (2018). Heterologous effects of infant BCG vaccination: potential mechanisms of immunity. Future Microbiol.

[4] Kaushal D (2015). Mucosal vaccination with attenuated Mycobacterium tuberculosis induces strong central memory responses and protects against tuberculosis. Nat Commun.

[5] White AD (2013). Evaluation of the safety and immunogenicity of a candidate tuberculosis vaccine, MVA85A, delivered by aerosol to the lungs of macaques. Clin Vaccine Immunol.

[6] Goonetilleke NP (2003). Enhanced immunogenicity and protective efficacy against Mycobacterium tuberculosis of bacille Calmette-Guerin vaccine using mucosal administration and boosting with a recombinant modified vaccinia virus Ankara. J Immunol.

[7] Giri PK, Verma I, Khuller GK (2006). Protective efficacy of intranasal vaccination with Mycobacterium bovis BCG against airway Mycobacterium tuberculosis challenge in mice. J Infect.

[8] Chen L, Wang J, Zganiacz A, Xing Z (2004). Single intranasal mucosal Mycobacterium bovis BCG vaccination confers improved protection compared to subcutaneous vaccination against pulmonary tuberculosis. Infect Immun.

[9] Shen Y (2002). Adaptive immune response of Vgamma2Vdelta2+ T cells during mycobacterial infections. Science.

[10] Shen L (2019). Immunization of Vgamma2Vdelta2 T cells programs sustained effector memory responses that control tuberculosis in nonhuman primates. Proc Natl Acad Sci USA.

[11] Dean GS (2015). Protection Induced by Simultaneous Subcutaneous and Endobronchial Vaccination with BCG/BCG and BCG/Adenovirus Expressing Antigen 85A against Mycobacterium bovis in Cattle. PLoS One.

[12] Whelan A (2012). Immunogenicity comparison of the intradermal or endobronchial boosting of BCG vaccinates with Ad5-85A. Vaccine.

[13] Lai R, Afkhami S, Haddadi S, Jeyanathan M, Xing Z (2015). Mucosal immunity and novel tuberculosis vaccine strategies: route of immunisation-determined T-cell homing to restricted lung mucosal compartments. Eur Respir Rev.

[14] Li W, Deng G, Li M, Liu X, Wang Y (2012). Roles of Mucosal Immunity against Mycobacterium tuberculosis Infection. Tuberc Res Treat.

[15] Manjaly Thomas ZR, McShane H (2015). Aerosol immunisation for TB: matching route of vaccination to route of infection. Trans R Soc Trop Med Hyg.

[16] Santosuosso M (2005). Mechanisms of mucosal and parenteral tuberculosis vaccinations: adenoviral-based mucosal immunization preferentially elicits sustained accumulation of immune protective CD4 and CD8 T cells within the airway lumen. J Immunol.

[17] Horvath CN, Xing Z (2013). Immunization strategies against pulmonary tuberculosis: considerations of T cell geography. Adv Exp Med Biol.

[18] Satti I (2014). Safety and immunogenicity of a candidate tuberculosis vaccine MVA85A delivered by aerosol in BCG-vaccinated healthy adults: a phase 1, double-blind, randomised controlled trial. Lancet Infect Dis.

[19] Horvath CN, Shaler CR, Jeyanathan M, Zganiacz A, Xing Z (2012). Mechanisms of delayed anti-tuberculosis protection in the lung of parenteral BCG-vaccinated hosts: a critical role of airway luminal T cells. Mucosal Immunol.

[20] Shaler CR, Horvath C, Lai R, Xing Z (2012). Understanding delayed T-cell priming, lung recruitment, and airway luminal T-cell responses in host defense against pulmonary tuberculosis. Clin Dev Immunol.

[21] Jeyanathan M, Heriazon A, Xing Z (2010). Airway luminal T cells: a newcomer on the stage of TB vaccination strategies. Trends Immunol.

[22] Mittrucker HW (2007). Poor correlation between BCG vaccination-induced T cell responses and protection against tuberculosis. Proc Natl Acad Sci USA.

[23] Majlessi L (2006). An increase in antimycobacterial Th1-cell responses by prime-boost protocols of immunization does not enhance protection against tuberculosis. Infect Immun.

[24] Salerno A, Dieli F (1998). Role of gamma delta T lymphocytes in immune response in humans and mice. Crit Rev Immunol.

[25] Hayday AC (2000). [gamma][delta] cells: a right time and a right place for a conserved third way of protection. Annu Rev Immunol.

[26] Hayday ACG (2009). T cells and the lymphoid stress-surveillance response. Immunity.

[27] Siddiqui N, Price S, Hope J (2012). BCG vaccination of neonatal calves: potential roles for innate immune cells in the induction of protective immunity. Comp Immunol Microbiol Infect Dis.

[28] Qaqish A (2017). Adoptive Transfer of Phosphoantigen-Specific gammadelta T Cell Subset Attenuates Mycobacterium tuberculosis Infection in Nonhuman Primates. J Immunol.

[29] Chen ZW, Letvin NL (2003). Adaptive immune response of Vgamma2Vdelta2 T cells: a new paradigm. Trends Immunol.

[30] McGill JL (2014). The role of gamma delta T cells in immunity to Mycobacterium bovis infection in cattle. Vet Immunol Immunopathol.

[31] Hoft DF, Brown RM, Roodman ST (1998). Bacille Calmette-Guerin vaccination enhances human gamma delta T cell responsiveness to mycobacteria suggestive of a memory-like phenotype. J Immunol.

[32] Chen CY (2013). Phosphoantigen/IL2 expansion and differentiation of Vgamma2Vdelta2 T cells increase resistance to tuberculosis in nonhuman primates. PLoS Pathog.

[33] Dieli F (2003). Differentiation of effector/memory Vdelta2 T cells and migratory routes in lymph nodes or inflammatory sites. J Exp Med.

[34] Gioia C (2002). Lack of CD27-CD45RA-V gamma 9V delta 2+ T cell effectors in immunocompromised hosts and during active pulmonary tuberculosis. J Immunol.

[35] Geginat J, Sallusto F, Lanzavecchia A (2003). Cytokine-driven proliferation and differentiation of human naive, central memory and effector memory CD4+ T. cells. Pathol Biol (Paris).

[36] Qin S (1998). The chemokine receptors CXCR3 and CCR5 mark subsets of T cells associated with certain inflammatory reactions. J Clin Invest.

[37] Glatzel A (2002). Patterns of chemokine receptor expression on peripheral blood gamma delta T lymphocytes: strong expression of CCR5 is a selective feature of V delta 2/V gamma 9 gamma delta T cells. J Immunol.

[38] Godkin AJ, Thomas HC, Openshaw PJ (2002). Evolution of epitope-specific memory CD4(+) T cells after clearance of hepatitis C virus. J Immunol.

[39] Maggioli MF, Palmer MV, Thacker TC, Vordermeier HM, Waters WR (2015). Characterization of effector and memory T cell subsets in the immune response to bovine tuberculosis in cattle. PLoS One.

[40] Meraviglia S, El Daker S, Dieli F, Martini F, Martino A (2011). gammadelta T cells cross-link innate and adaptive immunity in Mycobacterium tuberculosis infection. Clin Dev Immunol.

[41] Guerra-Maupome M, Palmer MV, Waters WR, McGill JL (2019). Characterization of γδ T Cell Effector/Memory Subsets Based on CD27 and CD45R Expression in Response to. Immunohorizons.

[42] Dieli F (2003). Characterization of lung gamma delta T cells following intranasal infection with Mycobacterium bovis bacillus Calmette-Guerin. J Immunol.

[43] de Bree C (2018). Bacillus Calmette-Guerin-Induced Trained Immunity Is Not Protective for Experimental Influenza A/Anhui/1/2013 (H7N9) Infection in Mice. Front Immunol.

[44] Endsley JJ (2009). The calf model of immunity for development of a vaccine against tuberculosis. Vet Immunol Immunopathol.

[45] Waters WR (2011). Tuberculosis immunity: opportunities from studies with cattle. Clin Dev Immunol.

[46] Waters WR (2009). Efficacy and immunogenicity of Mycobacterium bovis DeltaRD1 against aerosol M. bovis infection in neonatal calves. Vaccine.

[47] Whelan AO (2008). Evidence for enhanced central memory priming by live Mycobacterium bovis BCG vaccine in comparison with killed BCG formulations. Vaccine.

[48] Vordermeier HM (2009). Viral booster vaccines improve Mycobacterium bovis BCG-induced protection against bovine tuberculosis. Infect Immun.

[49] Hope JC (2011). Identification of surrogates and correlates of protection in protective immunity against Mycobacterium bovis infection induced in neonatal calves by vaccination with M. bovis BCG Pasteur and M. bovis BCG Danish. Clin Vaccine Immunol.

[50] Maggioli, M. F. *et al*. Application of Long-term cultured Interferon-gamma Enzyme-linked Immunospot Assay for Assessing Effector and Memory T Cell Responses in Cattle. *J Vis Exp*, e52833, 10.3791/52833 (2015). 10.3791/52833 PMC4544920 26275095

[51] Keating SM (2005). Durable human memory T cells quantifiable by cultured enzyme-linked immunospot assays are induced by heterologous prime boost immunization and correlate with protection against malaria. J Immunol.

[52] Millington KA, Gooding S, Hinks TS, Reynolds DJ, Lalvani A (2010). Mycobacterium tuberculosis-specific cellular immune profiles suggest bacillary persistence decades after spontaneous cure in untreated tuberculosis. J Infect Dis.

[53] Perdomo, C. *et al*. Mucosal BCG Vaccination Induces Protective Lung-Resident Memory T Cell Populations against Tuberculosis. *MBio* **7**, 10.1128/mBio.01686-16 (2016). 10.1128/mBio.01686-16 PMC5120139 27879332

[54] Blumerman SL, Herzig CT, Baldwin CL (2007). WC1+ gammadelta T cell memory population is induced by killed bacterial vaccine. Eur J Immunol.

[55] Worku S, Gorse GJ, Belshe RB, Hoft DF (2001). Canarypox vaccines induce antigen-specific human gammadelta T cells capable of interferon-gamma production. J Infect Dis.

[56] Ghazal P, Dickinson P, Smith CL (2013). Early life response to infection. Curr Opin Infect Dis.

[57] Hoft DF (2018). PO and ID BCG vaccination in humans induce distinct mucosal and systemic immune responses and CD4(+) T cell transcriptomal molecular signatures. Mucosal Immunol.

[58] Belyakov IM, Moss B, Strober W, Berzofsky JA (1999). Mucosal vaccination overcomes the barrier to recombinant vaccinia immunization caused by preexisting poxvirus immunity. Proc Natl Acad Sci USA.

[59] Maue AC (2005). Analysis of immune responses directed toward a recombinant early secretory antigenic target six-kilodalton protein-culture filtrate protein 10 fusion protein in Mycobacterium bovis-infected cattle. Infect Immun.

[60] Wangoo A (2005). Advanced granulomatous lesions in Mycobacterium bovis-infected cattle are associated with increased expression of type I procollagen, gammadelta (WC1+) T cells and CD 68+ cells. J Comp Pathol.

[61] Meraviglia S, Caccamo N, Salerno A, Sireci G, Dieli F (2010). Partial and ineffective activation of V gamma 9V delta 2 T cells by Mycobacterium tuberculosis-infected dendritic cells. J Immunol.

[62] Poggi A (2004). Migration of V delta 1 and V delta 2 T cells in response to CXCR3 and CXCR4 ligands in healthy donors and HIV-1-infected patients: competition by HIV-1 Tat. Blood.

[63] Ferrero E (2003). Macrophages exposed to Mycobacterium tuberculosis release chemokines able to recruit selected leucocyte subpopulations: focus on gammadelta cells. Immunology.

[64] Fuller CL, Flynn JL, Reinhart TA (2003). *In situ* study of abundant expression of proinflammatory chemokines and cytokines in pulmonary granulomas that develop in cynomolgus macaques experimentally infected with Mycobacterium tuberculosis. Infect Immun.

[65] Mikhak Z (2006). STAT1 in peripheral tissue differentially regulates homing of antigen-specific Th1 and Th2 cells. J Immunol.

[66] Palmer MV, Waters WR, Whipple DL (2002). Aerosol delivery of virulent Mycobacterium bovis to cattle. Tuberculosis (Edinb).

[67] Guerra-Maupome, M. *Characterization of bovine unconventional memory-like responses induced by Mycobacterium bovis infection and vaccination. Doctor of Philosophy Thesis, Kansas State University*, (2019).

