# Supplementary material for: Characterization of local and circulating bovine γδ T cell responses to respiratory BCG vaccination
Source: Sci Rep. 2019 Nov 5;9:15996. doi: 10.1038/s41598-019-52565-z (PMC6831659; doi:10.1038/s41598-019-52565-z)
Supplement: Supplementary file 1 — Supplemental Figures [file 41598_2019_52565_MOESM1_ESM.docx]

**Characterization of local and circulating bovine γδ T cell responses to respiratory BCG vaccination**

Mariana Guerra-Maupome^1^, Jodi L. McGill^1*^

^1^ Department of Veterinary Microbiology and Preventive Medicine, Iowa State University, Ames, IA, USA.

*Corresponding author:

Jodi L. McGill

Email: jlmcgill@iastate.edu


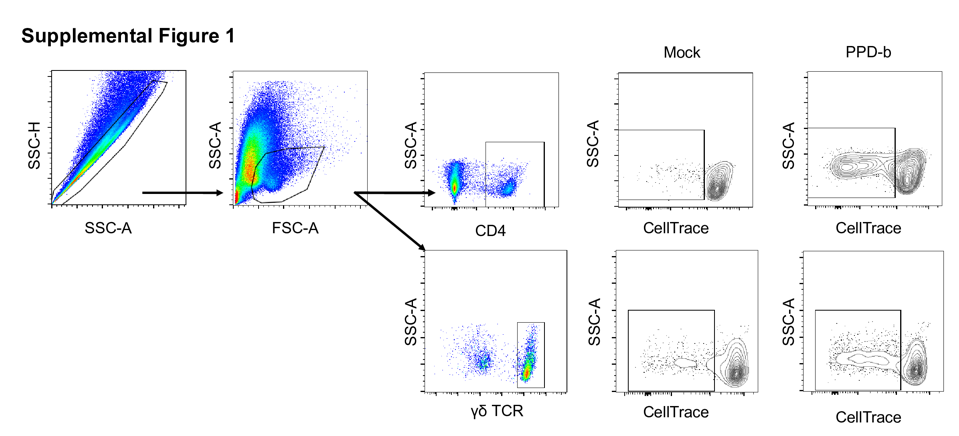


**Supplementary Figure 1. PBMC gating strategy.** PBMCs from control (n= 10) or BCG–vaccinated animals (n= 18) were labeled with CellTrace, and 5 × 10 ^6^ cells/ml were cultured for 6 days in the presence or absence of PPD-b. Cells were labeled with anti-bovine γδ TCR or CD4 and analyzed by flow cytometry for CellTrace dilution. Gating hierarchy (gating sequence as depicted by the arrows): Single cells (SSC-A vs SSC-H), lymphocytes (SSC-A vs FSC-A), γδ T cells or CD4 and CellTrace dilution. Representative contour plots of proliferative responses to *in vitro* PPD-b stimulation. Analysis was performed with Flowjo software.


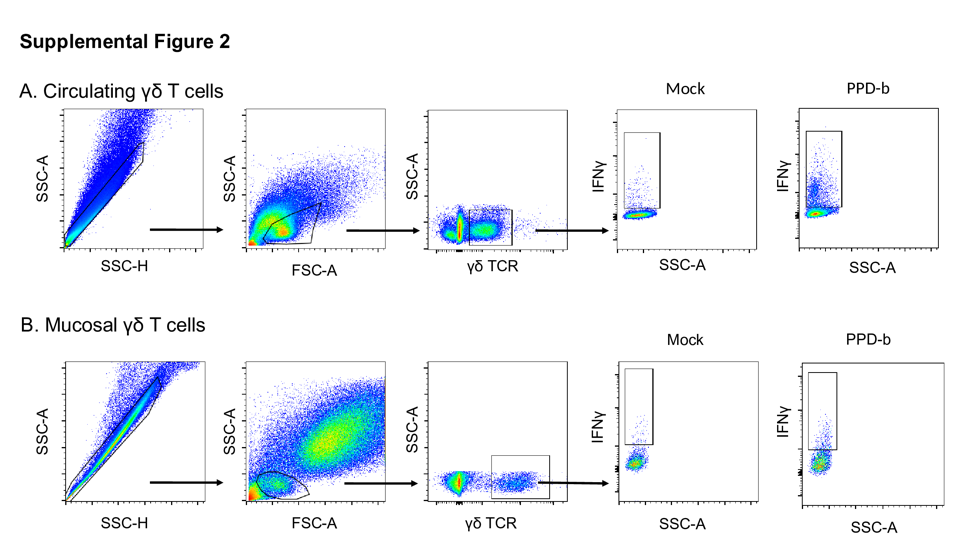


**Supplementary Figure 2. PBMC and BAL cells gating strategy.** Approximately ~8 weeks after aerosol vaccination, IFNγ expression was analyzed in circulating **(A, PBMC)** and in mucosal **(B, BAL)** compartment from control (n=7) or BCG–vaccinated animals (n= 7). 1 x 10 ^6^ cells/well were stimulated in vitro with PPD-b (200 IU/ml) for 16 hours. Cells were then stained for intracellular IFNγ expression and analyzed by flow cytometry. Gating hierarchy (gating sequence as depicted by the arrows): Single cells (SSC-A vs SSC-H), lymphocytes (SSC-A vs FSC-A), γδ T cells and IFNγ expression as shown. Analysis was performed with Flowjo software.


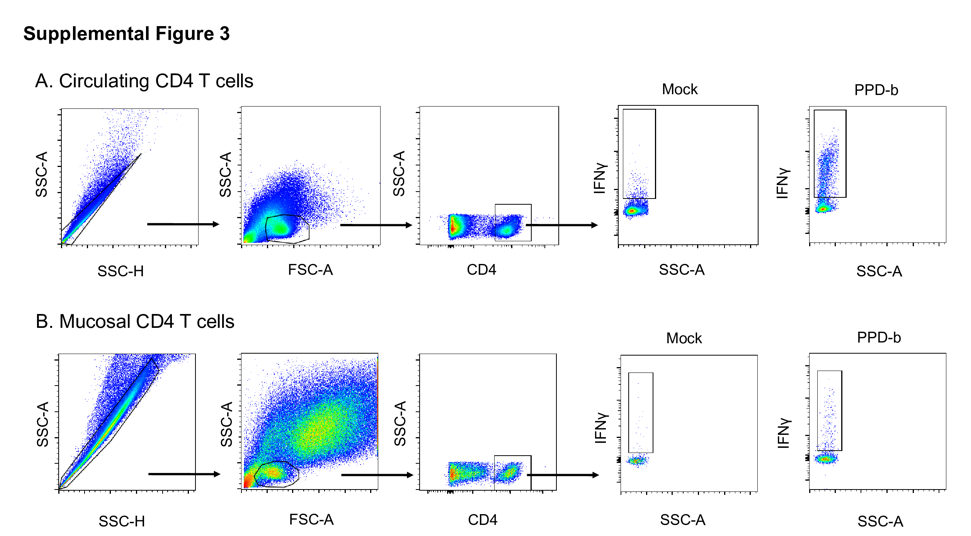


**Supplementary Figure 3. PBMC and BAL cells gating strategy.** Approximately ~8 weeks after aerosol vaccination, IFNγ expression was analyzed in circulating **(A, PBMC)** and in mucosal **(B, BAL)** compartment from control (n=7) or BCG–vaccinated animals (n= 7). 1 x 10 ^6^ cells/well were stimulated in vitro with PPD-b (200 IU/ml) for 16 hours. Cells were then stained for intracellular IFNγ expression and analyzed by flow cytometry. Gating hierarchy (gating sequence as depicted by the arrows): Single cells (SSC-A vs SSC-H), lymphocytes (SSC-A vs FSC-A), CD4 T cells and IFNγ expression as shown. Analysis was performed with Flowjo software.


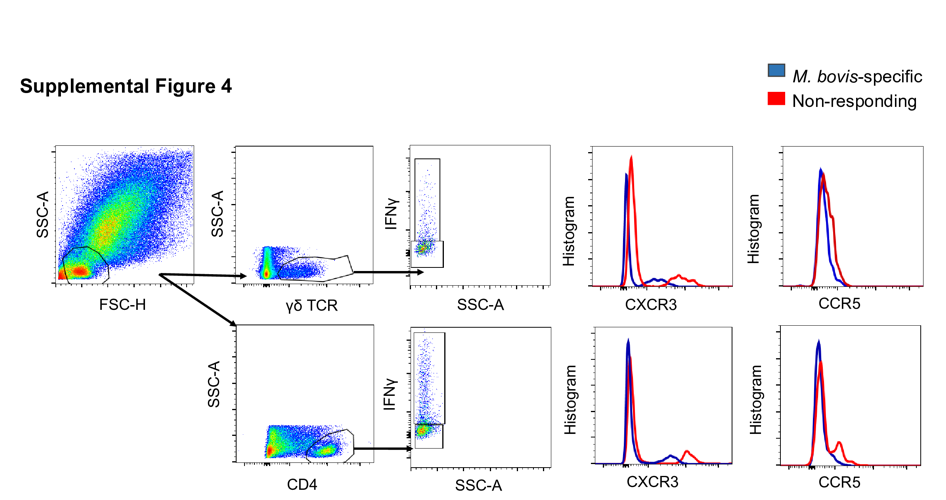


**Supplementary Figure 4. BAL cells gating strategy for** **expression of chemokine receptors on mucosal γδ and CD4 T cells.** BAL cells were isolated from calves ~8 weeks after vaccination. Cells were stimulated with PPD-b in vitro for 16 hours. Cells were surface stained and then analyzed by flow cytometry to study CXCR3 and CCR5 expression on mucosal M. bovis-specific γδ and CD4 T cells. Gating hierarchy (gating sequence as depicted by the arrows): Lymphocytes (SSC-A vs FSC-A), γδ T cells or CD4 cells, IFNγ+ and IFNγ^neg^ cells and CXCR3 or CCR5 expression as shown in histogram. Analysis was performed with Flowjo software.


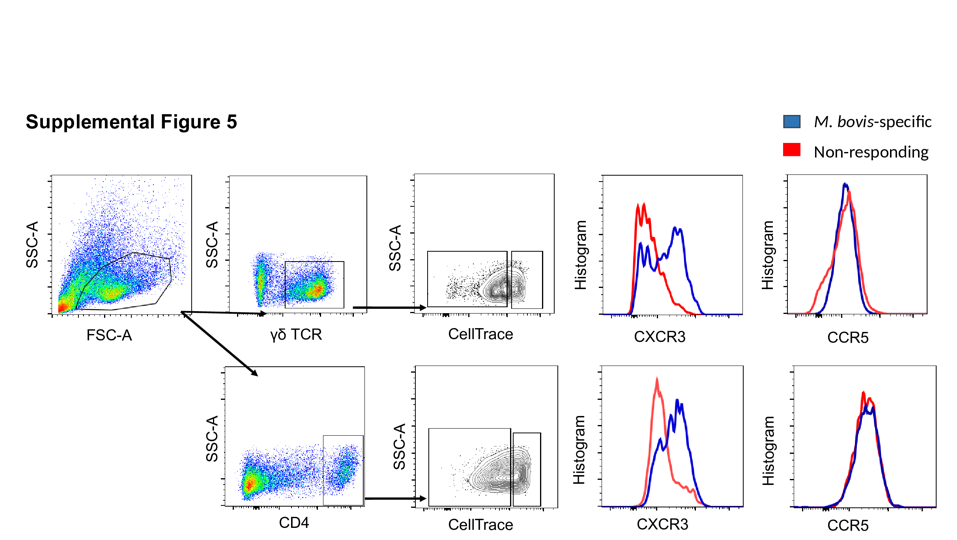


**Supplementary Figure 5. PBMC cells gating strategy for expression of chemokine receptors on mucosal γδ and CD4 T cells.** PBMCs were isolated from calves ~8 weeks after vaccination. Cells were stained with CellTrace dye and incubated with PPD-b for 6 days. Cells were surface stained and then analyzed by flow cytometry to study CXCR3 and CCR5 expression on circulating *M. bovis*-specific γδ and CD4 T cells. Gating hierarchy (gating sequence as depicted by the arrows): Lymphocytes (SSC-A vs FSC-A), γδ T cells or CD4 cells, proliferating and non-proliferating cells (CellTrace dilution) and CXCR3 or CCR5 expression as shown in histogram. Analysis was performed with Flowjo software.


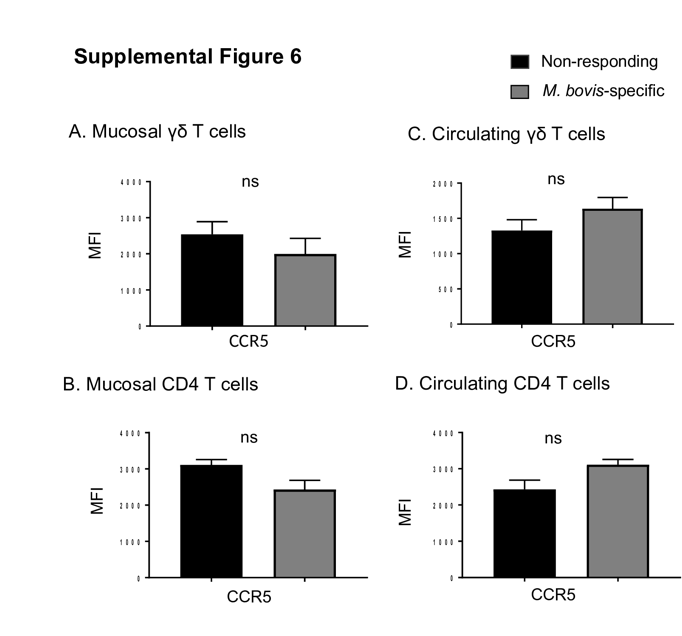


**Supplementary Figure 6. PBMC and BAL-recovered cells expression of chemokine receptors on mucosal γδ and CD4 T cells.** Cells were isolated from calves ~8 weeks after vaccination. Cells were stimulated in vitro as indicated in Materials and Methods, surface stained and then analyzed by flow cytometry to study CXCR3 and CCR5 expression on γδ and CD4 T cells. Analysis was performed with Flowjo software. Not significant (ns) difference from antigen-responsive cells compared to non-responsive cells as determined by Student’s t-test.
